# The Genomic Landscape of Serrated Lesion of the Colorectum: Similarities and Differences With Tubular and Tubulovillous Adenomas

**DOI:** 10.3389/fonc.2021.668466

**Published:** 2021-10-12

**Authors:** Luigi Tornillo, Frank Serge Lehmann, Andrea Garofoli, Viola Paradiso, Charlotte K. Y. Ng, Salvatore Piscuoglio

**Affiliations:** ^1^ Institute of Medical Genetics and Pathology, University Hospital Basel, Basel, Switzerland; ^2^ GILAB AG, Labor für Gastrointestinale Pathologie, Allschwil, Switzerland; ^3^ Division of Gastroenterology and Hepatology, University Hospital of Basel, Basel, Switzerland; ^4^ Department of Biomedicine, Visceral Surgery and Precision Medicine Research Laboratory, Basel, Switzerland; ^5^ Department for BioMedical Research, University of Bern, Bern, Switzerland; ^6^ Swiss Institute of Bioinformatics (SIB), Lausanne, Switzerland

**Keywords:** colorectal cancer, serrated lesions, DNA sequencing, *BRAF*, tubulovillous adenoma

## Abstract

Serrated lesions of the colorectum are the precursors of 15–30% of colorectal cancers (CRCs). These lesions have a peculiar morphological appearance, and they are more difficult to detect than conventional adenomatous polyps. In this study, we sought to define the genomic landscape of these lesions using high-depth targeted sequencing. Eight sessile serrated lesions without dysplasia (SSL), three sessile serrated lesions with dysplasia (SSL/D), two traditional serrated adenomas (TSA), and three tubular adenomas (TA) were retrieved from the files of the Institute of Pathology of the University Hospital Basel and from the GILAB AG, Allschwil, Switzerland. Samples were microdissected together with the matched normal counterpart, and DNA was extracted for library preparation. Library preparation was performed using the Oncomine Comprehensive Assay targeting 161 common cancer driver genes. Somatic genetic alterations were defined using state-of-the-art bioinformatic analysis. Most SSLs, as well as all SSL/Ds and TSAs, showed the classical *BRAF* p.V600E mutation. The *BRAF*-mutant TSAs showed additional alterations in *CTNNB1*, *NF1*, *TP53*, *NRAS*, *PIK3CA*, while TA showed a consistently different profile, with mutations in *ARID1A* (two cases), *SMAD4*, *CDK12*, *ERBB3*, and *KRAS*. In conclusion, our results provide evidence that SSL/D and TSA are similar in somatic mutations with the *BRAF* hotspot somatic mutation as a major driver of the disease. On the other hand, TAs show a different constellation of somatic mutations such as *ARID1A* loss of function.

## Introduction

Colorectal cancer (CRC) is one of the most frequent tumors worldwide (1). The development of CRC represents a classical example of carcinogenesis, with the adenoma-carcinoma sequence being a well-established model for several epithelial tumors (2). Multiple (epi)genetic alterations in the epithelial cells of the intestine lead to the development first of an adenoma, that in a minority of cases may transform into CRC (2). In the last 20 years, however, the landscape of colorectal carcinogenesis has been partially modified by the explosion of molecular biology techniques. CRC is nowadays seen as a heterogeneous disease (3, 4). While two-thirds of CRCs arise through the “classical” chromosomal instability pathway, 15–30% probably arise through the “serrated neoplasia pathway” (5–7). The molecular events underlying the development of serrated neoplasia have been partially unraveled. In general, CRC can be roughly classified according to the following criteria (1): *APC*-status (mutated or wild-type) (2); microsatellite status (stable or unstable, MSS or MSI) (3); *KRAS* status (mutated or wild-type) (4); *BRAF* status (mutated or wild-type) (5); methylation status (CpG Islands methylation phenotype high or low, CIMP-H or CIMP-L) (6, 8, 9). The most interesting and important topic is that the molecular and morphological heterogeneity of CRC corresponds to clinical heterogeneity (e.g., localization, prognosis, response to therapy). This has led to dramatic changes in the surveillance, prevention, and treatment of CRC (3).

The morphological features of the serrated pathway are the so-called serrated polyps. Currently, the WHO classification recognizes three major categories of serrated lesions, namely, the hyperplastic polyp (HP), the sessile serrated lesion (SSL, with or without dysplasia), and the traditional serrated adenoma (TSA), based mainly on the work of Torlakovic and Snover (9, 10). This classification is based on the distinct histological and cytological features such as morphology and the “serrated” (i.e., resembling “sawtooth”) architecture of colon crypts, and the position and the extent of the proliferative zone in the crypts (7, 11, 12). While the preneoplastic potential of HPs is still debated, “true” preneoplastic lesions are SSL and TSA. Because SSLss have a higher incidence, they can be viewed as the most important precursors of malignancy in the serrated pathway (12).

The morphology of the serrated pathway seems to match, at least partially, specific driver molecular alterations. The most frequent is the V600E mutation in the *BRAF* proto-oncogene (13, 14). Additional epigenetic changes as observed in the CIMP-H phenotype, namely, hypermethylation of the *MLH1* gene, may lead to microsatellite instability (7, 13, 14) and subsequent development of CRC. However, no complete correspondence exists between the CIMP-H phenotype and the serrated pathway (5). Additionally, the study by the Cancer Genome Atlas (TCGA) has identified six pathways that are altered in CRC (WNT, TGF-beta, RTK/RAS, PI3K, TP53, MS) (15), and more recently a molecular classification distinguishing four subgroups has been suggested (16).

In this study, we analyzed a series of histologically well-characterized precursor lesions of CRC by NGS to characterize the genetic landscape of serrated lesions and to compare it with tubular adenomas.

## Materials and Methods

### Patients and Tissue Samples

Eight sessile serrated lesions (SSL), three sessile serrated lesions with dysplasia (SSL/D), and two traditional serrated adenomas (TSA) were retrieved from the files of the Institute of Pathology of the University Hospital Basel, Switzerland, and from the GILAB AG, Allschwil, Switzerland, between January 1 and June 30, 2016. For all selected samples, a matched normal mucosa was available and was used as germline control. The histological classification for the samples is summarized in **Table 1**. Three tubular adenomas (TA) were used as control. All the slides were reviewed by an experienced pathologist (LT) to confirm the histological diagnosis, using the current WHO classification (9). The study has been approved by the Institutional Review Board of the Institute of Pathology, University Hospital Basel, and the Ethics Committee of Nordwest/Central Switzerland (EKNZ).

**Table 1:** Histologic and immunohistochemical characterization (mismatch repair proteins) of the samples included in the study.

Case ID	Diagnosis	MLH1	MSH2	MSH6	PMS2
SSA01T	SSL w/o dysplasia	Pos	Pos	Pos	Pos
SSA02T	SSL w/o dysplasia	Pos	Pos	Pos	Pos
SSA03T	TSA with serrated dysplasia, low-grade	Pos	Pos	Pos	Pos
SSA04T	TSA with serrated dysplasia, low-grade	Pos	Pos	Pos	Pos
SSA05T	SSL with adenomatous dysplasia, low-grade	Neg	Pos	Pos	Pos
SSA06T	SSL with adenomatous dysplasia, low-grade	Pos	Pos	Pos	Pos
SSA07T	SSL w/o dysplasia	Pos	Pos	Pos	Pos
SSA08T	SSL w/o dysplasia	Pos	Pos	Pos	Pos
SSA09T	SSL w/o dysplasia	Pos	Pos	Pos	Pos
SSA10T	SSL w/o dysplasia	Pos	Pos	Pos	Pos
SSA11T	SSL w/o dysplasia	Pos	Pos	Pos	Pos
SSA12T	SSL with adenomatous dysplasia, low-grade	Pos	Pos	Pos	Pos
SSA13T	SSL w/o dysplasia	Pos	Pos	Pos	Pos
SSA14T	TA, low-grade dysplasia	Pos	Pos	Pos	Pos
SSA15T	TA, low-grade dysplasia	Pos	Pos	Pos	Pos
SSA16T	TA, low-grade dysplasia	Pos	Pos	Pos	Pos

### DNA Extraction and Microdissection

Eight-μm-thick sections from representative formalin-fixed paraffin-embedded (FFPE) histological blocks for each lesion and matched normal mucosa were stained with Nuclear Fast Red in RNase-free conditions and subjected to microdissection with a sterile needle under a stereomicroscope (Olympus) to ensure a percentage of tumor cells >90%, as described previously (17). DNA was extracted from the microdissected tissues using the DNeasy Blood and Tissue Kit (Qiagen), according to the manufacturer’s guidelines, and quantified using the Qubit Fluorometer assay (Life Technologies). All samples yielded DNA of sufficient quantity and quality for sequencing analysis.

### Targeted Sequencing

Library preparation was performed using the Oncomine Comprehensive Assay v3 (Thermo Fisher Scientific) using 20 ng DNA according to the manufacturer’s guidelines. This multiplex-PCR based technique targets 161 common cancer driver genes. Quantification and quality control were performed with the Ion Library TaqMan Quantitation Kit (Thermo Fisher Scientific). Pooled libraries diluted to 30 pM each were loaded on Ion 530 Chip (Thermo Fisher Scientific) and processed in Ion Chef Instrument (Thermo Fisher Scientific). Sequencing was performed on the Ion S5 system (Thermo Fisher Scientific).

### Mutation Analysis

Somatic mutation calling was performed using PipeIT (18), which performs the initial variant calling step by Torrent Variant Caller (TVC, v5.0.3, Thermo Fisher Scientific) using low stringency variant calling parameters. PipeIT whitelists hotspot variants (19, 20) then filters out variants covered by fewer than 10 reads in either the tumor or the matched normal sample, supported by fewer than eight reads or for which the tumor variant allele frequency (VAF) was <10 times that of the matched normal VAF. Whitelisted hotspot variants that did not pass the above read count and/or VAF filters were manually reviewed. All *BRAF* V600E mutations were confirmed to be somatic by Sanger sequencing as previously described (21).

### Immunohistochemistry

Immunohistochemical staining was performed as previously described (22, 23). Briefly, 8 μm-thick sections were cut, dehydrated, and processed on a Bond III (Leica Biosystems Wetzlar, Germany). The ready-to-use (RTU) primary antibodies were from Novocastra (Leica Biosystems, Wetzlar, Germany), optimized for use on BOND III. The staining was performed with the following antibodies:

MLH1 (clone ES 05): Epitope retrieval (ER) 1, pH 7.5/8, 30’ 100°C; incubation time: 30’
MSH2 (clone 79H11): ER 2, pH9, 20’ 100°C; incubation time: 30’
MSH6 (clone EP 49): ER2, pH9, 20’ 100°C; incubation time: 30’
PMS2 (clone EP 51); ER2, pH9, 10’ 100°C; incubation time: 30’

## Results

### Mutational Analysis

**Figure 1:**
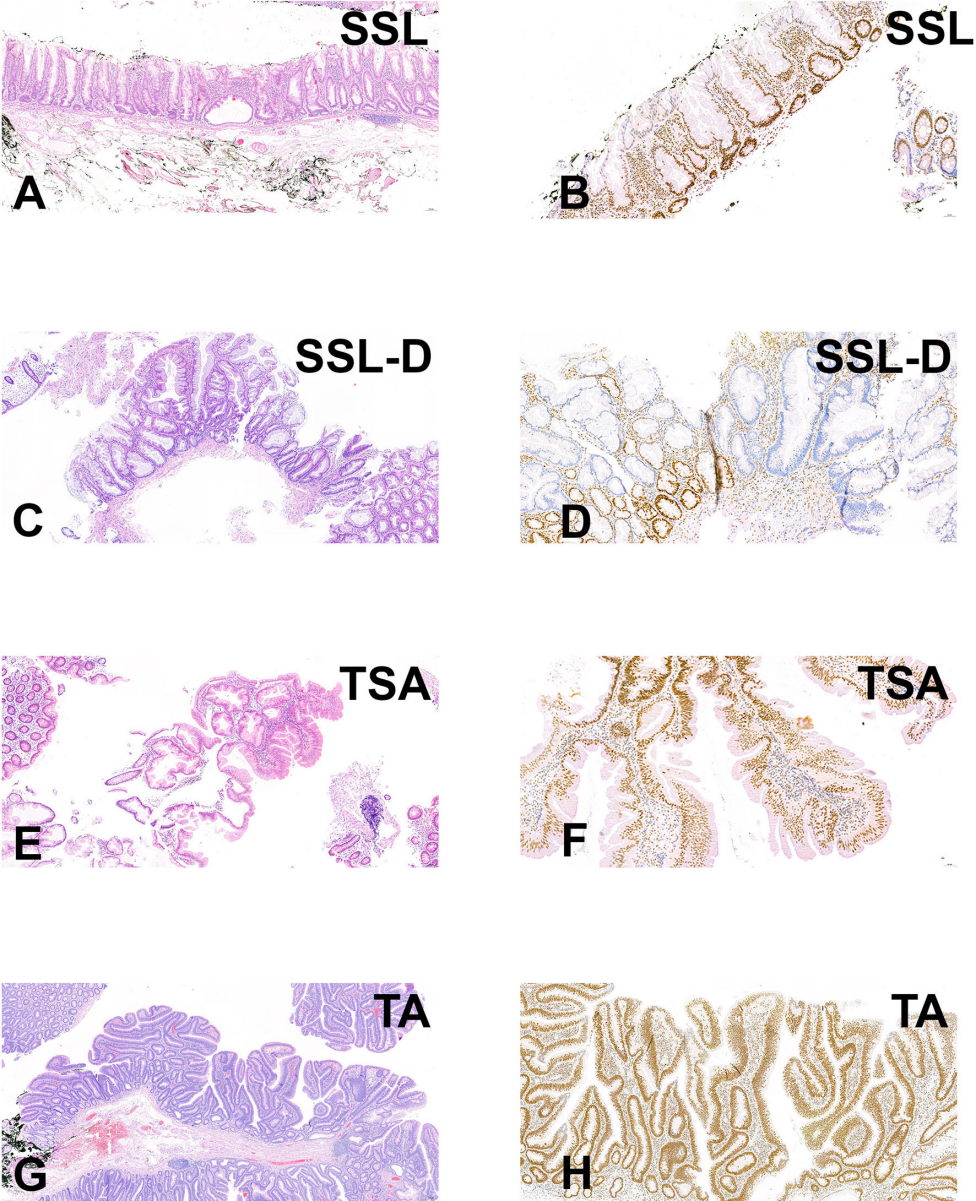
Histological micrographs **(A, C, E, G)** and immunohistochemical stain results for MLH1 **(B, D, F, H)**. **(A, B)** SSL without dysplasia (SSA08T); **(C, D)** SSL with dysplasia (ID SSA05T); **(E, F)** TSA (SSA04T); **(G, H)** TA (SSA15T). Note negativity for MLH1 in SSA05T **(D)**.

**Figure 2:**
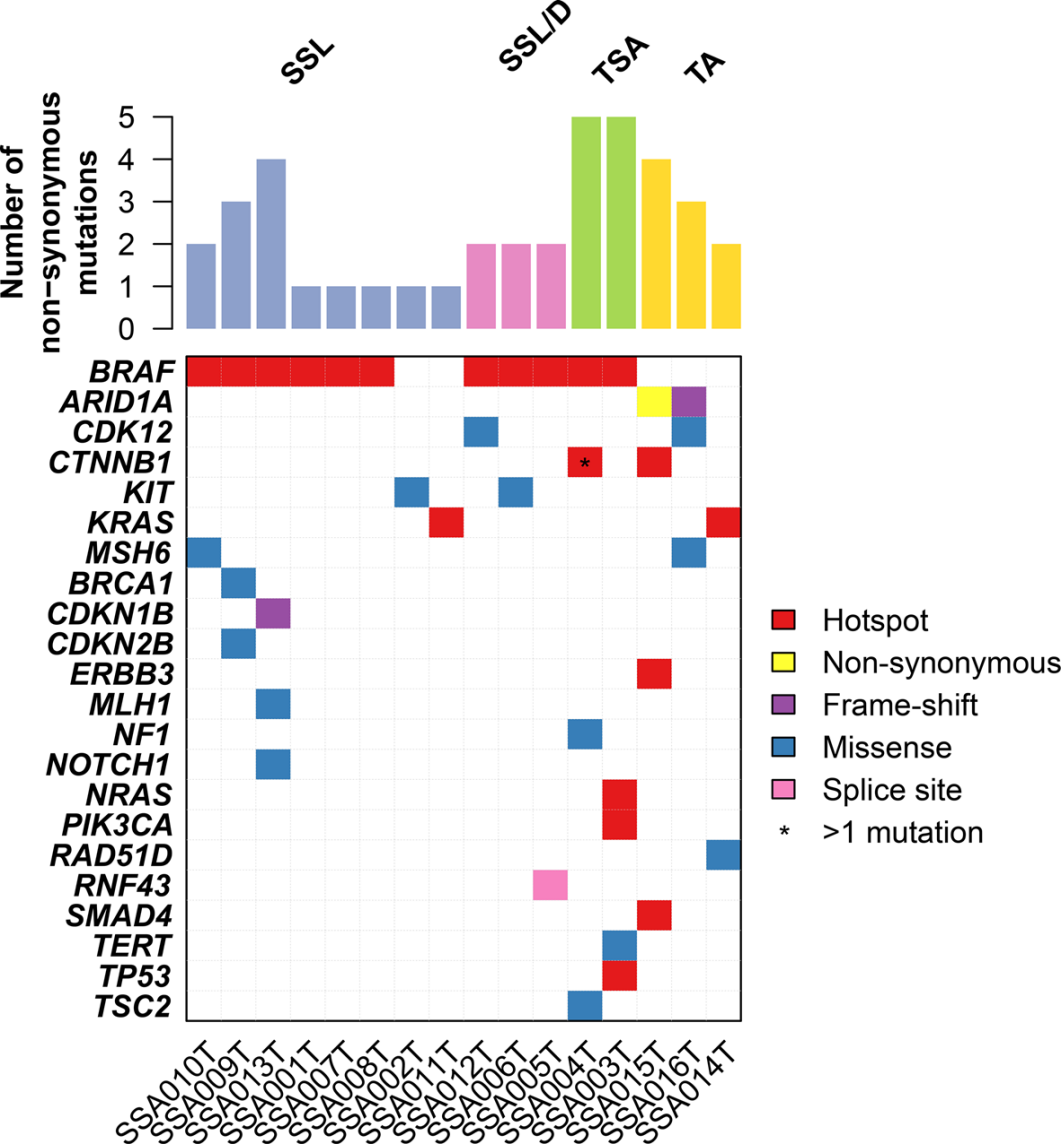
Repertoire of somatic genetic alterations in the serrated lesion of the colorectum. Heatmap indicating somatic genetic alterations identified in samples subjected to sequencing. Somatic alterations are color-coded according to the legend. Hotspot mutations are non-synonymous mutations that are in hotspot residues (see *Materials and Methods*).

We performed targeted sequencing of eight sessile serrated lesions without dysplasia (SSL), three sessile serrated lesions with dysplasia (SSL/D), two traditional serrated adenomas (TSA, **Figure 1**), as well as three tubular adenomas (TA) as control. Sequencing was performed at a mean depth of 670× (range from 382× to 942×; **Supplementary Table 1**). On average, SSLs harbored 1.75 mutations (range 1–4, n=8), SSL/Ds 2 mutations (range 2–2, n=3), TSAs 55 mutations (range 5–5, n=2), and TAs 3 mutations (range 2–4, n=3) (**Figure 2**). Annotation of the somatic mutations reveals that all but two samples harbored hotspot alteration in cancer-related genes.

All SSL/D and TSAs showed the classical *BRAF* p.V600E mutation, together with 75% (6/8) of SSLs. Of note, one of the two *BRAF*-wildtype SSLs harbored a hotspot *KRAS* Q61K mutation (**Figure 2** and **Supplementary Table 2**). Additionally, the *BRAF*-mutant TSAs showed additional alterations in *CTNNB1*, *NF1*, *TP53*, *NRAS*, *PIK3CA*.

We further identified alterations involving *MLH1* and *MSH6*, part of the “mismatch repair” machinery, in two SSLs. However, these mutations were missense rather than truncating, and their variant allele fractions were ~5%, suggesting they may not result in mismatch repair deficiency. Indeed, immunohistochemistry of MLH1, MSH2, MSH6, and PMS2 showed that all SSLs were positive, indicating mismatch repair proficiency (**Figure 1** and **Table 1**). On the other hand, we identified one SSL/D (SSA005T) that was MLH1-negative.

On the other hand, TAs showed a consistently different profile and were all wild-type for BRAF (p = 0.02, Fisher’s exact test compared to the serrated lesions). By contrast, TAs harbored mutations in *ARID1A* (two cases), *SMAD4*, *CDK12, ERBB3*, and *KRAS*. The complete list of mutations is shown in **Supplementary Table 2**.

Taken together, our results provide evidence that SSL/Ds and TSAs are similar in somatic mutations having the *BRAF* hotspot somatic mutations as a major driver of the disease. On the other hand, TAs show a different constellation of somatic mutations such as *ARID1A* loss of function.

## Discussion

CRC is the third neoplasia for incidence and the fourth cause of death for neoplasia worldwide (1). Although CRC represents the classical model of development of epithelial cancer through the so-called adenoma-carcinoma sequence (2), it is now clear that there are other genetic events underlying its origin and growth. In particular, the clinical and biological relevance of serrated lesions of the colon has been pointed out in the last 25 years (5, 6). Serrated lesions of the colon are very well defined from the pathological point of view (12), and patients with serrated lesions are followed up similarly to “classical” tubular (TA), tubulovillous (TVA), and villous adenomas (VA) of the colon.

In general, in our series, only TSA showed a higher frequency of mutations, while SSL, TA, and TVA had fewer mutations/cases. This is in agreement with a recent study, in which no difference was found between SSL and conventional adenomas regarding the frequency of mutations (24). Although we only have two TSAs in our series, the presence of five mutations/case in comparison to ≈1.75–2 in SSL or TA may suggest that genomic instability could underlie the morphology of TSA.

We found the classical mutation *BRAF* p.V600E in most serrated lesions in our series, in particular in all SSLs with dysplasia and TSAs. SSLs with dysplasia are considered the “true” premalignant lesion to CRC in the so-called “serrated pathway” and progress relatively quickly, whereas TSAs are more similar to “conventional” adenomas regarding the development of cancer (25, 26). *BRAF* is one of the key players in the development of CRC through serrated adenomas (27, 28). *BRAF* is a molecular switch in the RAS–RAF–MEK–ERK pathway, which is crucial in the control of proliferation and differentiation (28). In our study, we found *KRAS* mutations in 1/4 conventional adenomas and in 1/12 serrated polyps (SSL without dysplasia, *BRAF*-wildtype), which is in keeping with the results of several previous studies, where the frequency of *KRAS* mutations in both conventional and serrated adenomas was between 8 and 10% (29–32). *BRAF* mutation has been suggested to be an early phenomenon in the genesis of SSL, whereas the genetic landscape of TSA seems to be more heterogeneous (4). In a recent study using whole-exome sequencing, *BRAF* V600E was found to be the only consistent mutation in serrated polyps (33). In our study, two SSLs without dysplasia had no *BRAF* mutation. One case had a p.Q61K mutation in *KRAS*, and the other had a p.A507P substitution in *KIT*. The former is a rare mutation that has been described in non-small-cell lung cancer and in colon cancer, with oncogenic potential. The latter has been described in GISTs as oncogenic.

Other mutations found in SSLs affected *BRCA1*, a very important tumor suppressor gene, involved in the genesis and development of many different tumors (34), *CDKN1B* and *CDKN2B* (also known as p27^KIP1^ and p15^INK4B^), two important tumor suppressor genes involved in the regulation of cell cycle. We also found two SSLs with mutations affecting proteins of the DNA-repair machinery (*MLH1* and *MSH6*). Immunohistochemistry showed that all SSLs were mismatch repair proficient, thus these *MLH1* and *MSH6* mutations are not likely to have affected the mismatch repair pathway, and it is also unlikely that these SSL polyps were driven by *MLH1* methylation. Similarly, *NOTCH1* has been shown to act as an oncogene in CRC (35), so it is likely that the missense mutation we found is simply a bystander phenomenon.

SSL/D had a monotonous genomic landscape. They showed the classical *BRAF* V600E mutation, as described in the literature (5, 33, 36). They also showed mutations in *CDK12* and in *RNF43*. *CDK12* is a recently characterized cyclin-dependent kinase (CDK) that has been claimed to be involved in the genesis of prostate and ovarian cancers (37). *RNF43* belongs to a superfamily of thrombospondin type 1 repeat-containing proteins (R-spondins) (38). R-spondins control the activity of WNT signaling also in adult stem cells (38). Interestingly, missense mutations in *RNF43* have been found consistently in microsatellite unstable CRCs (39) and in some hereditary serrated polyposes (40). It is however a well-known factor in the development of gastric adenocarcinoma (41) and pancreatic intraductal papillary mucinous neoplasm (42). We also identified one SSL/D with loss of MLH1 expression without *MLH1* somatic mutations. One could speculate here that the dysplasia, in this case, was driven by *MLH1* methylation.

TSA showed in contrast more mutations (five/case). *BRAF* p.V600E was found in both cases. Other mutations were found in *CTNNB1* (beta-catenin), *NF1*, *TSC2*, *NRAS*, *PIK3CA*, *TERT*, *TP53*. The role of beta-catenin in Wnt signaling and colon carcinogenesis is very well known (15). It has been shown that *TSC2*, through its interaction with mTOR, may increase the activity of Wnt signaling (43). Moreover, there is a cross-talk between *PIK3CA* and Wnt signaling, probably again mediated by TSC2 (43). *NF1* changes have not been described previously in serrated lesions. Interestingly, it was claimed to be a target of mutational changes in MSI-CRCs (44). *TERT* is a well-known factor involved in the progression of colorectal carcinogenesis and higher activity (45). p.Q61K mutation in *NRAS* is a well-known activating mutation, with oncogenic potential. p.C275Y missense mutation in *TP53* has been found in colorectal cancer (15), and mutations in *TP53* are associated with the development of dysplasia in TSAs (46).

TAs showed three mutations/case, but they were more variable. One case showed the classical *KRAS* p.G12C. Other involved genes were *ARID1A*, *CDK12*, *CTNNB1*, *KIT*, *MSH6*, *ERBB3*, *RAD51D*. Truncating mutations of *ARID1A*, a chromatin remodeler, have been related to the development of endometrioid carcinoma of the ovary (47) and of early CRC (48). *ERBB3* belongs to the HER receptor tyrosine kinase (RTK) family and has been found to be mutated in 11% of CRCs (49). *RAD51D*, involved in DNA repair, has been found to be rarely mutated in ovarian, breast, and colon cancer (50). Of note, our sequencing panel does not include *APC*, which is an important cancer gene in the conventional pathway. A previous study found that *APC* mutations are rare in serrated lesions, which would suggest *APC* mutations may be another distinguishing molecular feature that distinguishes the conventional and the serrated pathways (51).

The main limitation of our work is that it is a small retrospective series. The use of a targeted sequencing panel also limits our ability to characterize the global genomic features of these lesions. The study of the methylation profiles of these samples could also provide additional insights into the pathogenesis of the serrated pathway. Further study in larger cohorts will be required to confirm our results.

In summary, we have studied by NGS a small series of colorectal serrated lesions (SSL, SSL/D, and TSA) and have compared their genomic profiles with conventional TAs. Serrated lesions have a simpler genomic profile in comparison with TAs. *BRAF* p.V600E is the most frequent genomic alteration in serrated lesions.

## Data Availability Statement

Sequencing data are be available on the European Genome-Phenome Archive database under the accession number EGAS00001005648.

## Ethics Statement

The studies involving human participants were reviewed and approved by the Ethics Committee of Nordwest/Central Switzerland (EKNZ). The patients/participants provided their written informed consent to participate in this study.

## Author Contributions

LT and SP conceived and supervised the study. LT performed the histologic review. AG performed the bioinformatics analyses under the supervision of CN. VP extracted the DNA and performed the sequencing reactions. LT, FL, LMT, CN, and SP analyzed the data and critically discussed the results. LT, CN, and SP wrote the manuscript. All authors contributed to the article and approved the submitted version.

## Funding

The study was supported by grants from Oncosuisse KFS-4988-02-2020-R to SP. SP was also supported by the Theron Foundation, Vaduz (LI) and from the Surgery Department of the University Hospital Basel. The funding bodies had no role in study design; in the collection, analysis, and interpretation of data; in the writing of the report; and in the decision to submit the article for publication.

## Conflict of Interest

The authors declare that the research was conducted in the absence of any commercial or financial relationships that could be construed as a potential conflict of interest.

## Publisher’s Note

All claims expressed in this article are solely those of the authors and do not necessarily represent those of their affiliated organizations, or those of the publisher, the editors and the reviewers. Any product that may be evaluated in this article, or claim that may be made by its manufacturer, is not guaranteed or endorsed by the publisher.

## Supplementary Material

Supplementary Table 1 Sequencing statistics.

Supplementary Table 2 Somatic single nucleotide variants (SNVs) and insertion/deletions (indels) identified in our cohort.
